# Responsive policies needed to secure rural supply from increasing female doctors: A perspective

**DOI:** 10.1002/hpm.3363

**Published:** 2021-10-15

**Authors:** Belinda O'Sullivan, Matthew McGrail, Jennifer May

**Affiliations:** ^1^ Faculty of Medicine The University of Queensland Rural Clinical School Toowoomba Queensland Australia; ^2^ Faculty of Medicine The University of Queensland Rural Clinical School Rockhampton Queensland Australia; ^3^ Department of Rural Health (UONDRH) The University of Newcastle Tamworth New South Wales Australia

**Keywords:** female doctors, gender, policy, recruitment, rural medicine

## Abstract

Around the world, the supply of rural health services to address population health needs continues to be a wicked problem. Adding to this, an increasing proportion of female doctors is graduating from medical courses but gender is not accounted for within rural workforce policy and planning. This threatens the future capacity of rural medical services. This perspective draws together the latest evidence, to make the case for industry and government action on responsive policy and planning to attract females to rural medicine. We find that the factors that attract female doctors to rural practice are not the same as males. We identify female‐tailored policies require a re‐visioning of rural recruitment, use of employment arrangements that attract females and re‐thinking issues of rural training and specialty choice. We conceptualise a roadmap that includes co‐designing rural jobs within supportive teams, allowing for capped hours which align with childcare along with boosting of female peer support and mentorship. There is also a need to enhance flexible rural postgraduate training options in a range of specialties (at a time when many women are establishing families) and to consider viable partner employment (including for female doctors with university trained partners) and advertising specific rural attractors to women, including the chance to connect with communities and make a difference.

## 1 INTRODUCTION

Worldwide the proportion of females taking up careers in medicine is increasing. Females now outnumber males in medical school graduations in most high and some low‐ and middle‐income countries.^1^, ^2^, ^3^ In Australia, females were 35% of pre‐2000 graduates rising to 53% for post‐2000 graduates.^4^ Key impacts have been forecast including increased difficulty filling full time jobs, on‐call rosters and leadership roles, as well as an oversupply of graduates seeking people‐focused specialties with plannable working hours.^1^, ^2^, ^5^, ^6^, ^7^, ^8^, ^9^, ^10^, ^11^, ^12^ However, the potential impacts of gender on rural supply have been ignored within international health policy, planning and management to date. The World Health Organization's (WHO) global policy recommendations concerning developing, attracting, recruiting, and retaining the health workforce in rural and remote areas (updated in 2021) are gender‐neutral, although they go as far as to suggest there is a need for more research on outcomes of female health workers in rural communities.^13^ There are signs that the gender‐neutrality that currently exists across strategies is causing a critical failure of rural policies, therefore wasting scarce national and regional resources and restricting access of rural people to female doctors.^4^, ^14^ This perspective aims to explore the nuance of female rural medical workforce policy and planning to argue for a roadmap to improve female uptake and retention in rural medicine.

The potential implications of limited female uptake of rural medicine are widespread, given the worldwide shortage of rural doctors. Key national studies in the United Kingdom, United States, Japan and Australia over the last 20 years identify lower rural work participation by female doctors,^6^, ^15^ their higher rural turnover^16^ and lower uptake of rural generalist specialties,^15^ but there remains little action on gender‐related workforce distribution issues by industry and government.

Perhaps it is easier to assume that rural medicine and its broader skill set, multisite practice models, longer and more on‐call hours is simply at odds with female work expectations and therefore unmodifiable.^17^, ^18^, ^19^, ^20^, ^21^ However, there has been minimal analysis of the existing evidence to inform responsive and globally transferrable policies, to secure rural supply from the increasing numbers of female doctors. In this perspective, we identify concerns with mainstream recruitment, employment, and training systems and how they could be adjusted to better accommodate female requirements.^13^, ^22^, ^23^ Our perspective identifies three themes for guiding future action, including re‐visioning rural recruitment, using employment arrangements that attract females, and attending to rural training and specialty choice to make them attractive to females. We then suggest how these pieces could fit together for solutions of international relevance.

## 2 METHOD

Three authors with long‐standing academic experience of studying medical workforce development, particularly for rural populations, drew purposefully from their practice networks and existing global literature bases to source any policy and research material related to medical workforce distribution where gender had been explored as a covariate or key study factor. Snowballing was done from the reference list of articles and by purposefully searching on websites to source further relevant material. Using experts to source this material was considered appropriate as a formal literature review method was thought to have limitations in finding material where stratification of evidence by gender was not the main topic. Given the aim of this paper was to develop a perspective of female‐specific rural medical workforce issues, the material was iteratively organized with respect to informing policy. To do this, three authors met regularly to discuss the policy themes. This was done until the authors agreed the final themes.

## 3 RESULTS

The key policy themes are described: re‐visioning rural recruitment, using employment arrangements that attract females and rural training and specialty choice.

### 3.1 Theme 1: re‐visioning rural recruitment

Female doctors build their careers alongside of distinctive life events. Having young dependent children reduces the total hours worked by female general practitioners (GPs) and specialists, but not males.^24^, ^25^ This decrease is largest for female GPs (who may work nearly 12 h less per week if they have children aged 0–4 years).^25^ This does not rebound to the same lifetime hours as men. UK data suggests female doctors provide 25% less lifetime hours of employee contribution than male doctors.^1^ Specific to rural areas, despite more female than male doctors employed in one Australian state (2004–2012), they contributed 41% less hours compared with male counterparts in rural areas.^26^ The service gap is even wider when reviewing workloads of retiring rural doctors, who currently provide a wide service portfolio of emergency on call services projected to require 1.25 male or 1.4 female ‘next‐generation’ doctors to maintain the same level of service.^27^

To re‐vision rural recruitment to the volume of full time equivalent (FTE) male and female doctors needed, it is critical to explore the reasons why females are attracted to rural work. Female rural family physicians in the United States enjoyed the extended scope of work, loyalty and leadership of colleagues and excellent back up support in rural practice.^28^ They connected with rural areas if they had a rural background and enjoyed a sense of belonging (doctor and partner) and feeling that they were making a difference,^29^ strengthened through building relationships with rural patients and families.^30^

The partners of female doctors play a key role in influencing career behaviour. A US study suggested partner employment was very significant for female rural physicians compared with male.^31^ Female rural GPs in Australia moved to larger towns if their partner was seeking employment whereas this had no effect on male GPs.^32^ Supporting a partner's work can also draw female doctors to rural areas—around 40% of female rural GPs followed their partner's rural job to end up in rural practice.^33^ Partner work also needs to consider a partner's qualification levels. A study of Norwegian doctors found females were more likely to have university‐qualified partners than male doctors, thus limiting their ability to work rurally (87% and 37%, respectively).^34^

The detractors of females pursuing rural medicine also need to be addressed. Female rural doctors may be deterred by social or professional isolation and lack of privacy in rural areas.^30^, ^31^ Females may also be concerned about whether a rural practice environment can support any work transitions such as taking maternity leave, or the risk of additional workload they might encounter from failed workforce recruitment to fill service gaps.^28^ An Australian study showed that as soon as female GPs have children, they are more likely to leave rural practice as opposed to males who tended to leave when their children reached secondary school ages.^32^ Overall, this suggests holistically focused recruitment strategies need to accommodate work, life and partner factors for GPs and specialists in order to attract and keep female doctors in rural areas.

### 3.2 Theme 2: using employment arrangements that attract females

There is limited evidence about the nature of rural employment encouraging of female participation but examples can be drawn from the wider literature about female medical labour. A Norwegian study identified that females employed in salaried hospital roles with children aged 0–5 years were just as likely to work full time compared with all doctors, as males in similar roles.^34^ The reasons have not been researched. It could be due to better access to local, affordable and fit‐for‐purpose childcare options. Alternatively it may indicate that structured and predictable hours assist females to manage family responsibilities for young children, just as easily as male counterparts. This concurs with other research showing that female, compared with male specialists, are more likely to work exclusively in public sector roles (39% and 25%, respectively).^24^ Studies identify that specialists working in the public sector also incur less weekly working hours than those in mixed practice roles (both public and private work).^24^ Also, working in larger hospital teams (possible in some specialties) may provide some relief from on call demands which could be attractive to females with children.^34^

To attract female doctors with children to rural areas, viable childcare and schooling options that blend well with employment models may also be important. Furthermore, evidence suggests that rural towns which provide salaried roles and collaborative team‐based styles of working, may be more attractive for females, over competitive roles that operate on fee for service bases.^35^

Additional evidence about female doctors' decision‐making identifies that they desire sustainable employment options, including factors like being able to work closer to home with limited job‐related travel requirements.^36^ It is eminently possible to structure rural medical positions with these parameters in mind, so as to attract more females.

### 3.3 Theme 3: rural training and specialty choice

A central pillar of rural workforce development is to *grow your own* through selecting rurally oriented medical students who can pursue rural training, although rural training strategies remain gender neutral, rather than tailored to females.^13^ One localised programme found that medical students who had regular rural experience and mentorship increased uptake of rural work by female and male graduates at similar levels, up to postgraduate year 15, relative to doctors who had no rural training.^37^ An Australian study also found (significantly) more females participate in rural undergraduate medical training (70%) compared with males (56%).^4^ However, there may be gaps in the postgraduate interventions as female graduates were 20%–40% less likely than males to work rurally after they completed the medical degree.^4^ The reasons as to why, remain poorly explored.

Structured rural vocational training programmes, with clear rotations, mentorship and recognised training endpoints may attract more female medical graduates; one rural generalist programme with these aspects did achieve gender parity of enrolments.^26^ Other evidence reinforces that rural medical role models may assist females with rural career decision‐making and achieving a supported practice model.^5^, ^38^, ^39^ However, structured rural vocational training programmes across generalist fields needed in rural areas are relatively rare globally, noting there is a strong example in one province of Canada.^40^ The sparsity of integrated rural vocational training options in countries like Australia, the United Kingdom and the United States is a potential detractor for retaining female rural doctors across the range of specialties that rural areas need. This is a prominent issue given many high‐income countries are witnessing an increasing proportion of medical graduates pursuing non‐general practice specialties.^41^ If non‐GP specialty doctors are required to be in metropolitan areas for vocational training, at a time when many females are having children, it becomes increasingly unlikely they will uproot their lives again to work rurally once they are qualified specialists.

Whilst there is a trend for more female doctors to choose general practice as their specialty, potentially a positive for increasing rural supply, most female GPs do not work rurally.^41^ This may occur because female choice of GP is for a balanced lifestyle^42^ and this may directly conflict with the perceptions of rural GPs working more hours of an unpredictable workload.^43^ US data reveal a similar pattern, whereby female graduates represent a higher proportion of more recent primary care specialists than past cohorts, but relatively fewer working rurally.^15^ UK evidence also suggests that females may also be more likely to switch to general practice careers at the crossover period in their late 20s to early 30s, typically during childbearing years,^11^ but this period is potentially incongruent with relocating for rural practice due to the abundance of social ties that female doctors may have at this point.

Akin to the evidence about recruitment and employment that is tailored to rural medicine, training options also need to enable fixed rosters, part‐time hours and working in larger, supportive team environments to attract females to train rurally.^5^, ^38^, ^39^ There are major gaps in this, even within Australia's general practice training policy which although having a rural training option, all trainees are required to commence at a full‐time load, with minimal flexibility to drop back to part‐time hours.^44^

**FIGURE 1:**
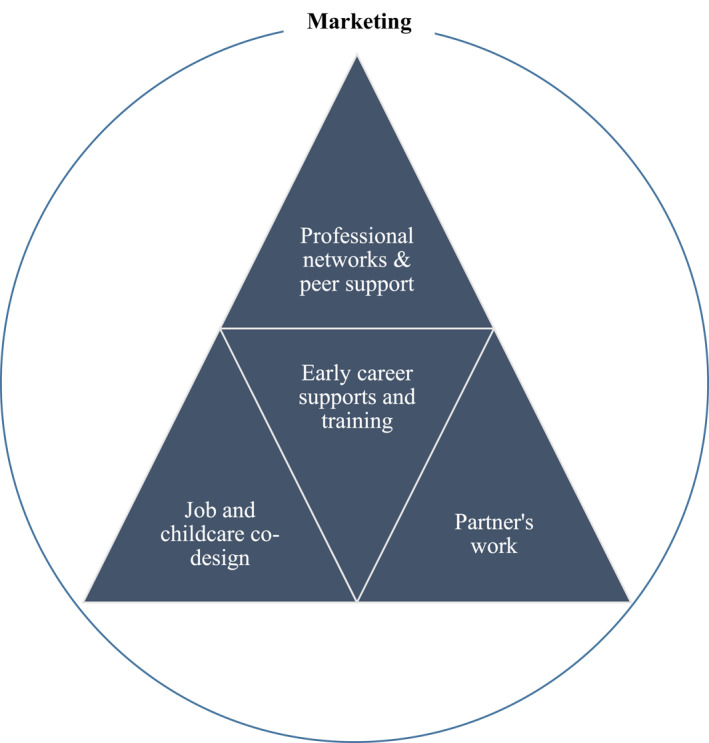
Fitting the pieces together to secure rural supply from the increasing number of female doctors

As nations face the critical challenge of improving access to rural medical services, it is essential to adjust policies, models and actions to target the needs of the increasing proportion of female doctors in the medical workforce. By drawing together the available evidence (Table 1), key solutions become apparent (Figure 1).

**TABLE 1:** Summary of attractors and detractors for females working in rural medicine

Considerations	Attractors	Detractors
Re‐visioning rural recruitment	Enough doctors to cover workload.	Sense of social and professional isolation and lack of privacy.
	Clinical teams that have leadership and clinical back up.	Lack of quarantined time for maternity leave and parenting
	Enjoy wider scope of work.	Fear of burden from failure of staff recruitment to fill service gaps.
	Sense of belonging (doctor and partner) in the community—including rural connections or experience.	Lack of help (social network) with children (newborn+)
	Sense of making a difference to community and building relationships with patients.	
	Communities which offer strong employment options for partners including if partner is university qualified.	
Employment arrangements	Jobs design that aligns with childcare/school hours (predictable).	Competitive roles in small teams
	Jobs allowing for part‐time hours and leave periods (flexible).	Few child minding options aligned with employment expectations
	Collaborative team‐based practice models.	Ongoing responsibility ‐ time off means further burden of work for others.
	Salaried roles.	
	Manageable on call requirements	
	Sustainable employment conditions allowing females to work close to home with limited travel, if desired.	
Rural training and specialty choice	Structured vocational training basing female doctors in rural areas at the time when many are having children.	Choice of general practice (GP) occurring later, at a time of having children when female doctors less likely to relocate.
	Female rural career mentors and colleagues.	Few rural and part‐time vocational training options in a range of relevant specialties available.

## 4 DISCUSSION

The findings can be assimilated into a roadmap to promote female attraction to rural medicine.

### 4.1 Marketing

Rural medicine could be better promoted to females as a chance to build relationships with people and make a difference to a community. This requires a shift from ‘deficit’ messaging to a ‘rural strengths’, more likely to hit home for those female doctors with previous experience or connections to rural communities. This will only lead to recruitment if all the desired job attributes are embedded through organisational commitment, and partner and childcare/schooling options have been considered.^45^ This is feasible to implement if there is greater awareness and engagement with these attractors as part of every rural marketing and recruitment campaign.

### 4.2 Professional networks and peer support

To mitigate negative impacts of professional isolation, it is essential to implement professional networks that connect rural female doctors working in similar fields regardless of where they work. This could happen at a global scale if online systems are adopted, removing barriers of distance. If regular and collaborative, these networks could build to forming a community of practice where people share tacit knowledge in an area of mutual interest, a learning process called ‘thinking together’.^46^ Also, employing multiple females, such as in rural job‐share roles, may improve informal peer support and comradery that is female specific. To minimise social isolation, female rural doctors could also be invited to local social events, granted travel time to visit other regions and major cities, and potentially given time off for local family visits. These interventions may be low‐cost relative to the cost of failed recruitment and retention, and may therefore be relatively feasible to implement. They go beyond mainstream WHO global recommendations about addressing personal and professional needs of health workers, to having specificity on the sense of professional and social connection that female doctors are likely to find affirming.

### 4.3 Job and childcare co‐design

First, rural medical jobs can be better structured for female doctors. This includes building and advertising practices or organisations with a sufficient critical mass of staff, cohesive teams, practice leadership, peer support, flexible work and leave options and manageable hours. Rather than designing roles that are shorter‐term, intense and destined for female doctor burnout, employees across a regional catchment could build sustainable and flexible roles with predictable hours, thereby having more potential to attract and retain female doctors.

Second, job design is only the beginning when females evaluate work feasibility around family time and childcare options. In particular rural childcare options should be regularly evaluated in relation to the demands of rural medical work, including managing unpredictable and antisocial hours, the need to accommodate different aged children, and the juggle of dual professional couples with or without other family or friends to support them.

Some of these suggestions depend on rural regions evaluating their strengths and opportunities to build sustainable and predictable rosters, including considering the availability of reliable childcare services which may need to be pursued through community and health service partnerships over a longer term. Once again, these suggestions are nuanced to female professional needs, building on the existing WHO global recommendations.^13^

### 4.4 Partners

Whilst the broader evidence around recruitment and retention to rural jobs largely ignores partner needs, the employment needs of partners plays a more prominent role in attracting and retaining rural female doctors. Rather than traditional recruitment processes being very individually focused, it may be better if rural communities adopted ‘whole‐of‐person’ recruitment and appraised how well their location could accommodate the needs of the families of female doctors. This may include expanding tools like the Community Apgar Questionnaire which rural communities can use to screen for factors related to doctor recruitment and thereby plan more nuanced recruitment strategies. This tool includes screening for spousal satisfaction, but this could more closely explore the fit of a place to the employment needs of female partners.^23^

In some rural areas, partner employment options may be hard to build in sufficient volume; however, in others, expanding partner jobs is possible including by working with local districts to aggregate parts of positions and leveraging ‘work from home’ options. Given that female medical partners are often university educated, they may be drawn to places with good Internet connections, community office hubs, short commutes and regular travel opportunities (airport and train) for professional collaboration. Once again, these suggestions are more nuanced to female doctor's personal needs than the strategoes proposed within the existing WHO global recommendations.^13^

### 4.5 Early career supports and training

Delivering a wider range of high quality structured vocational training in rural areas may assist to attract a female workforce in early career. Earlier attraction will have flow on effects as it draws females in at a time when they are having children, with partners who are also at formative career stages. This aligns with existing global evidence that ‘grow your own’ rural pathways could be more efficient for retention than relying on attracting doctors after they finished training in metropolitan locations.^47^

Second, training design and recruitment should account for any flexible hours needed by female doctors, mindful of the challenges of addressing the healthcare demands of rural communities. This may require more part‐time and job‐share prevocational and vocational roles as well as considerations of government subsidised paid maternity leave. This extends existing WHO global recommendations to promote training and work conditions that suit doctor's needs.

Finally, and importantly, selected female mentors could support and guide interested early career female doctors right through their careers, with rural‐specific career decision‐making. To work well, these programs should be well‐structured (based on reputable clinical leaders and sound governance), signposted and highly tailored to a range of female rural medical careers. These may be feasible to implement at low cost given the potential to draw from enthusiastic female doctors willing to volunteer and avail of low‐cost networking technologies. Professional support and networks are a key WHO global recommendation for rural workforce development; however, there is room for making these more female specific.^13^

## 5 CONCLUSION

To address responsive policies for increasing the supply of rural doctors we reviewed the latest evidence. We identify that the factors that attract females to rural medicine are not the same as males. We found that female‐tailored rural policies require a re‐visioning of traditional views of rural recruitment, applying employment arrangements specifically attractive to females and re‐thinking of rural training and specialty choice issues. We conceptualise the roadmap forward should include co‐designing rural jobs within supportive teams. These could be set around capped hours which align with childcare. There are potential gains from boosting female peer support and mentorship as well as enhancing flexible postgraduate training options in rural areas in a range of specialties (at a time when many women are establishing families). Viable partner employment is essential (including catering for university trained partners). The specific attractors for women to work in rural medicine, including the chance to connect with communities and make a difference should be leveraged. Responsive gendered policy and planning will capitalise on the attractors and mitigate detractors for female doctors pursuing rural medicine, thereby improving tailored recruitment, employment and specialty training towards a strong rural female medical workforce.

## CONFLICT OF INTEREST

There was no support from any organisation for the submitted work. The researchers who completed this work were employed under the Australian government's Rural Health Multidisciplinary Training Program, but had full independence for producing this analysis. There is no financial relationships with any organisations that might have an interest in the submitted work in the previous three years, and no other relationships or activities that could appear to have influenced the submitted work.

## ETHICS STATEMENT

This work did not require ethical approval as it did not involve primary data collection involving humans or animals.

## AUTHOR CONTRIBUTIONS

Belinda O'Sullivan and Matthew McGrail conceived of the study and collected the data. All authors contributed to analysis. Belinda O'Sullivan wrote the manuscript, and Matthew McGrail and Jennifer May contributed to the editing.

## Data Availability

Not applicable.
